# Water Extract of *Fritillariae thunbergii* Bulbus Inhibits RANKL-Mediated Osteoclastogenesis and Ovariectomy-Induced Trabecular Bone Loss

**DOI:** 10.3390/molecules27010169

**Published:** 2021-12-28

**Authors:** Ki-Shuk Shim, Dong-Ryun Gu, Youn-Hwan Hwang, Hyun Yang, Jin-Ah Ryuk, Hyunil Ha

**Affiliations:** 1 Herbal Medicine Research Division, Korea Institute of Oriental Medicine, Yuseong-daero 1672, Daejeon 34054, Korea; angeloshim@kiom.re.kr (K.-S.S.); mrwonsin@kiom.re.kr (D.-R.G.); hyhhwang@kiom.re.kr (Y.-H.H.); hyunyang@kiom.re.kr (H.Y.); yukjinah@kiom.re.kr (J.-A.R.); 2 Korean Convergence Medicine Major KIOM, University of Science & Technology (UST), Daejeon 34054, Korea

**Keywords:** *Fritillariae thunbergii* bulbus, osteoclast differentiation, ovariectomy, osteoporosis

## Abstract

*Fritillariae thunbergii* bulbus has been widely used to treat symptoms of coughs and airway congestion in the chest due to pathological colds and damp phlegm in traditional Chinese medicine. Despite its long history of traditional use, its pharmacological activities on osteoclastogenesis and osteoporosis have not been evaluated. This study investigated the effects of the water extract of *Fritillariae thunbergii* bulbus (WEFT) on osteoclast differentiation in bone marrow-derived macrophage cells and on ovariectomy (OVX)-induced osteoporosis in mice. We found that WEFT significantly inhibited osteoclastogenesis by downregulating the receptor activator of the NF-κB ligand (RANKL) signaling-induced nuclear factor of activated T-cells cytoplasmic 1 (NFATc1) expression. In an OVX-induced osteoporosis model, WEFT significantly prevented the OVX-induced trabecular loss of femurs, accompanied by a reduction in fat accumulation in the bone marrow and liver. In addition, WEFT significantly prevented weight gain and gonadal fat gain without recovering uterine atrophy. Using ultrahigh-performance liquid chromatography-tandem mass spectrometry, seven alkaloids (peimisine glucoside, yibeissine, peiminoside, sipeimine-glucoside, peimisine, peimine, and peiminine) were identified in WEFT. The results of this study suggest that WEFT can be a potential pharmacological candidate to reduce menopausal osteoporosis and menopause-related symptoms, such as fat accumulation.

## 1. Introduction

Osteoporosis is a bone disease characterized by a decrease in bone volume and bone mineral density, a gradual shortage of calcium, and an imbalance in bone remodeling [1]. Bone remodeling is the restructuring process of bone in adults to maintain bone homeostasis, which is the resorption of old or damaged bone accompanied by new bone formation [2,3]. The excessive activation of osteoclasts disrupts the net balance of bone remodeling, regressing bone microstructure and decreasing bone mineral density, which increases the progression of osteoporosis and eventually leads to a high incidence of bone fracture [4,5].

Receptor activator of NF-κB ligand (RANKL), generated from various cells, including stromal cells, osteoblasts, and osteocytes, is a pivotal cytokine that regulates osteoclast differentiation and activation [5]. RANKL/RANK signaling is initiated by the activation of tumor necrosis factor receptor-associated factor 6 (TRAF6) to stimulate MAPK, NF-κB, and AP-1 activation during the early phase of osteoclast differentiation [6,7]. It sequentially triggers the induction and activation of nuclear factor of activated T-cells cytoplasmic 1 (NFATc1) to regulate osteoclast-specific gene expression, such as tartrate-resistant acid phosphatase (TRAP) [8,9]. There are some negative regulators of NFATc1 at the transcriptional level, including v-maf avian musculoaponeurotic fibrosarcoma oncogene homolog B (MafB) and interferon regulatory factor-8 (Irf-8), which repress NFATc1 expression in osteoclast precursors [10,11]. Regarding the significant role of osteoclasts in bone remodeling, pharmaceutical studies to regulate osteoclastogenesis or its activation have been extensively investigated to develop effective anti-osteoporosis therapy [1].

*Fritillariae thunbergii* bulbus has been widely used as an ingredient in herbal formulas to treat lung diseases in traditional Chinese medicine [12]. Various pharmacological activities of *F. thunbergii* bulbus, including anti-tussive effects [13], tracheobronchial relaxation [14], anti-muscarinic activity [15], and anti-inflammatory activities [16] have been reported. In addition, phytochemical studies have identified various phytochemical components, such as alkaloids (peimine, peimisine, and sipeimine), essential oils, and diterpenoids in *F. thunbergii* bulbus [17,18]. However, the pharmacological effects of *F. thunbergii* bulbus on osteoporosis and osteoclast differentiation have not been studied. Therefore, the present study explored the anti-osteoporosis effects of the water extract of *F. thunbergii* bulbus (WEFT) in a mouse ovariectomy (OVX) model and the inhibitory mechanism of WEFT on osteoclast differentiation using bone marrow-derived macrophage cells (BMMs).

## 2. Results and Discussion

### 2.1. WEFT Inhibits RANKL-Induced Osteoclast Differentiation

**Figure 1:**
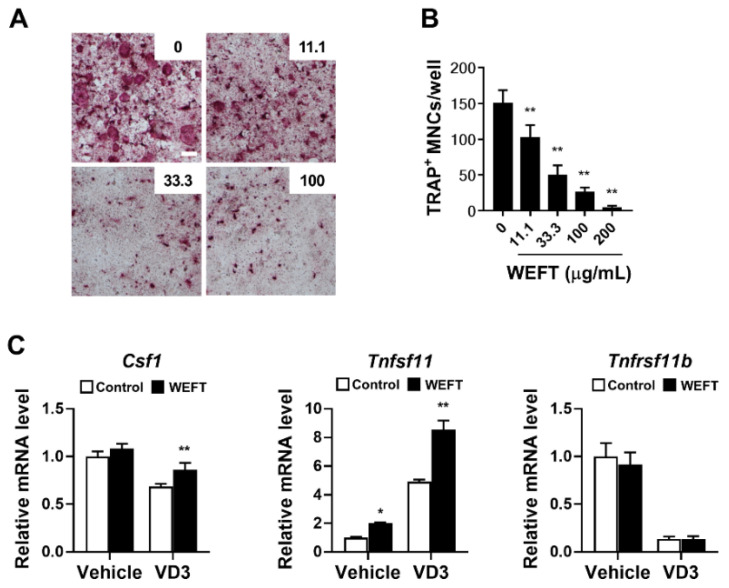
WEFT inhibits osteoclast differentiation of BMM co-cultured with MLO-Y. (**A**,**B**) BMMs were co-cultured with MLO-Y4 cells. 1α,25-dihydroxyvitamin D3 (VitD3, 10 nM) and different concentrations of WEFT (11.1, 33.3, 100, and 200 µg/mL) were treated in culture medium for five consecutive days. Typical images of TRAP-stained cells (**A**); (scale bar, 200 µm). The number of TRAP-positive multinucleated cells (TRAP^+^ MNCs) (**B**). (**C**) The mRNA expression of Csf1, Tnfsf11, and Tnfsf11b was analyzed using a quantitative real-time polymerase chain reaction (qPCR) after 1 day of incubation of MLO-Y4 cells with or without WEFT (100 µg/mL) and VitD3 (10 nM). * *p* < 0.05; ** *p* < 0.01 vs. vehicle.

Osteocytes are the major cells that produce RANKL for osteoclastogenesis in bone remodeling [19]. Therefore, we employed an osteocyte-BMM co-culture system using MLO-Y4 cells to examine the pharmacological effects of WEFT on osteocyte-dependent osteoclast differentiation. MLO-Y4, an osteocyte-like cell, supports the osteoclast differentiation of bone marrow precursor cells under co-culture conditions [20]. MLO-Y4 cells induce the osteoclast differentiation of precursors by RANKL binding, but also produce soluble RANKL under stress conditions [20,21]. As shown in Figure 1A,B, WEFT significantly and dose-dependently inhibited osteoclast differentiation in co-culture.

**Figure 2:**
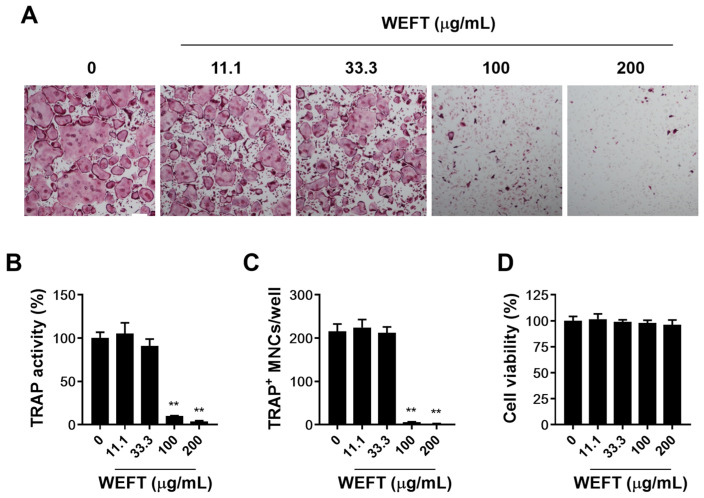
WEFT inhibits the RANKL-induced osteoclast differentiation of BMMs. BMM cultures were incubated with vehicle (distilled water) or WEFT (11.1, 33.3, 100, and 200 µg/mL) in the presence of M-CSF (60 ng/mL) and RANKL (50 ng/mL) for four consecutive days. (**A**) TRAP-stained cells were photographed (scale bar, 200 µm). (**B**) TRAP activities were examined and represented as the percentage of control. (**C**) TRAP-stained MNCs were enumerated. (**D**) BMMs were incubated with the indicated concentrations of WEFT for 24 h, and cell viability was measured by using a CellTiter-Glo luminescent cell viability assay. ** *p* < 0.01 versus vehicle.

However, when we examined the effects of WEFT on the mRNA expression of Csf1, Tnfsf11, and Tnfsf11b in MLO-Y4 cells, WEFT significantly increased the mRNA expression of Csf1 and Ttnfsf11 (Figure 1C) in VitD3-treated cells. This suggests that WEFT could directly inhibit osteoclastogenesis by acting on osteoclast precursors independently of its indirect effect on MLO-Y4 cells. Thus, we investigated the possible effects of WEFT on RANKL-induced osteoclast differentiation using BMM single cultures. We found that WEFT at 100 and 200 μg/mL markedly inhibited the RANKL-induced osteoclast differentiation of BMMs (Figure 2A), which was supported by WEFT’s inhibition of TRAP activity and osteoclast number (Figure 2B,C). WEFT did not affect BMM proliferation at the tested doses (Figure 2D). These results suggest that WEFT directly inhibited RANKL-induced osteoclast differentiation without cell toxicity.

### 2.2. WEFT Inhibits RANKL-Induced NFATc1 Expression

**Figure 3:**
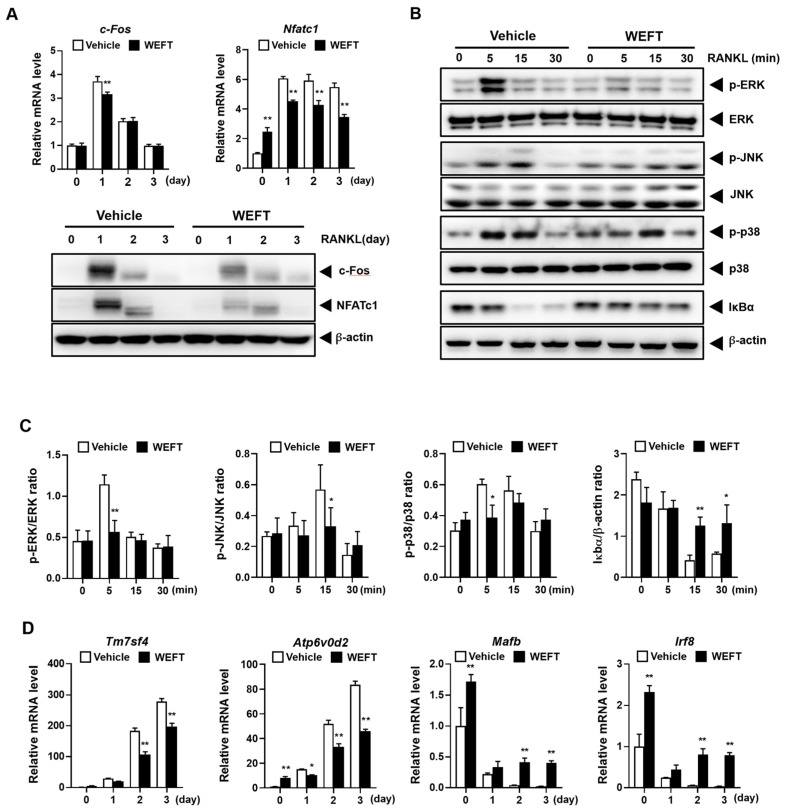
WEFT inhibits RANKL signaling pathway. (**A**) Vehicle or WEFT (100 µg/mL) was treated in BMM cultures at day 0 in the presence of M-CSF (60 ng/mL) and RANKL (50 ng/mL). The RANKL-induced mRNA or protein levels of c-Fos and NFATc1 were evaluated by qPCR or Western blot at day 0, 1, 2, and 3. (**B**) BMMs were stimulated with RANKL for the indicated minutes, 3 h after pre-treatment with WEFT (100 µg/mL). The indicated proteins were detected by Western blot. (**C**) Each phosphorylated MAPK level and total IκBα level were quantified by densitometry and normalized to total each MAPK and β-actin levels, respectively. (**D**) BMMs were treated as in A, and the mRNA expression of c-Tm7sf4, Atp6v0d2, Mafb, and Irf8 was analyzed using qPCR at day 0, 1, 2, and 3. * *p* < 0.05, ** *p* < 0.01 vs. vehicle.

RANKL signaling initiates TRAF6 to NFATc1 activation through MAPK, AP-1, and NF-κB signaling. MAPK, AP-1, and NF-κB transcriptionally induce NFATc1 expression in response to RANKL at an early phase of osteoclast differentiation [6,8]. NFATc1 also leads to the robust self-amplification of NFATc1 in cooperation with these molecules [22]. Therefore, we investigated the effect of WEFT on these components via qPCR and Western blot analysis. We selected 100 µg/mL of WEFT, the lowest concentration required to strongly inhibit osteoclast differentiation without cell toxicity. WEFT (100 µg/mL) significantly inhibited RANKL-induced c-Fos and NFATc1 expression at the transcriptional and translational levels (Figure 3A). WEFT significantly inhibited the RANKL-induced early activation of MAPK (ERK, JNK, and p38), determined by the phosphorylation levels. It also inhibited the RANKL-induced degradation of IκBα (Figure 3B,C). This suggests that WEFT significantly inhibits the RANKL signaling axis during the early phase of osteoclast differentiation. Based on WEFT’s inhibition on NFATc1 expression, we further evaluated NFATc1 target genes, Tm7sf4 and Atp6v0d2, which function in the cell fusion of osteoclast precursors during the late phase of osteoclast differentiation [23,24]. We found that WEFT significantly inhibited the mRNA levels of Tm7sf4 and Atp6v0d2 (Figure 3D), consistent with the decrease in multinuclear osteoclasts (Figure 2). RANKL can stimulate NFATc1 expression not only by up-regulating positive regulators but also by downregulating negative regulators. Therefore, we examined the mRNA expression of Mafb and Irf8, which are known to occupy the NFATc1 promoter region to suppress NFATc1 expression [10,11]. We found that WEFT significantly induced their expression at day 0 and suppressed the decrease induced by RANKL (Figure 3D), which might contribute to the suppression of RANKL-stimulated NFATc1 expression. These results suggest that WEFT inhibits osteoclastogenesis by suppressing the RANKL-induced positive and negative regulation of NFATc1 expression.

### 2.3. WEFT Attenuates OVX-Induced Bone Loss in Mice

**Figure 4:**
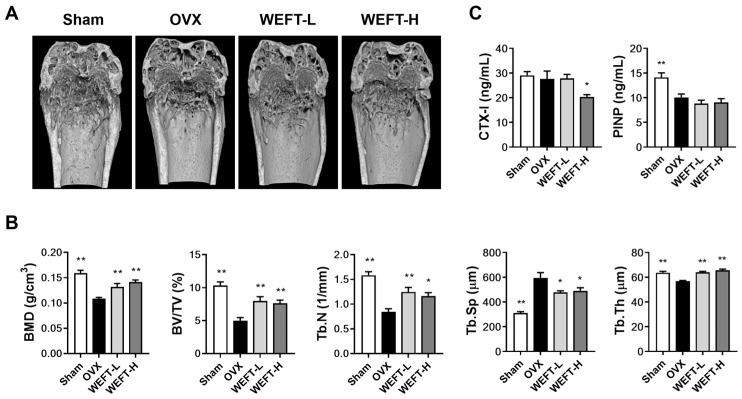
WEFT inhibits OVX-induced trabecular bone loss. Femora of OVX or sham mice were isolated and fixed in 10% neutral formalin after 6 weeks of sample administration (WEFT-L, 100 mg/kg/day; WEFT-H, 300 mg/kg/day) following sham or OVX. (**A**) Typical µ-CT images of trabecular bone in the distal femur of each group. (**B**) BMD and four microstructural parameters in trabecular bone were analyzed for quantification. (**C**) Serum levels of CTX-I and PINP were measured. BMD, bone mineral density; BV/TV, bone volume per tissue volume; Tb.N, trabecular number; Tb.Sp, trabecular separation; Tb.Th, trabecular thickness; CTX-I, C-terminal cross-linked telopeptides of type I collagen; PINP, procollagen type I N-terminal propeptide. * *p* < 0.05, ** *p* < 0.01 vs. OVX group.

Given WEFT’s inhibition of RANKL-induced osteoclastogenesis using BMMs, the in vivo pharmacological potential of WEFT was subsequently investigated using an OVX mouse model. This is a sex steroid deficiency-induced osteoporosis model with a significant disruption of trabecular microstructure and a decrease in bone mineral density (BMD) [25,26]. After 6 weeks of WEFT administration, micro-computed tomography (μ-CT) analysis of the femur revealed a remarkable trabecular loss in OVX mice compared with sham mice (Figure 4A). However, this was reverted by WEFT administration at a low dose (100 mg/kg/day, WEFT-L) and a high dose (300 mg/kg/day, WEFT-H). When BMD and four parameters of bone microstructure were analyzed and quantified, WEFT-L increased BMD by approximately 22%, bone volume per tissue volume (BV/TV) by 61%, trabecular number (Tb.N) by 48%, and trabecular thickness (Tb.Th) by 13%, but decreased trabecular separation (Tb.Sp) by 20% compared to the OVX group (Figure 4B). There was no statistical difference observed between WEFT-L and WEFT-H, suggesting that this is the optimal concentration of WEFT inhibition in OVX-induced osteoporosis. Estrogenic deficiency upregulates RANKL expression by osteocytes or stromal cells, which increases bone resorption activity and accelerates bone turnover rate [27]. Estrogenic deficiency also diminishes osteoblastic Fas ligand-induced apoptosis of osteoclasts, thereby increasing osteoclast survival [28]. To elucidate the in vivo effects of WEFT on bone resorption and formation, we measured the serum concentrations of C-terminal cross-linked telopeptides of type I collagen (CTX-I, a marker of bone resorption) and procollagen type I N-terminal propeptide (PINP, a marker of bone formation). At the end of the experimental period, OVX mice displayed lower PINP levels than sham mice, whereas the CTX-I levels were similar to sham mice. WEFT-H decreased CTX-I levels without affecting PINP levels compared to the OVX group (Figure 4C). These results suggest that the inhibition of osteoclast-mediated bone resorption might mainly contribute to the bone protective effect of WEFT. However, the precise mechanisms underlying the anti-osteoporotic effect of WEFT remain to be further elucidated because, unlike WEFT-H, WEFT-L inhibited OVX-induced bone loss without affecting CTX-I levels at the experimental endpoint.

**Figure 5:**
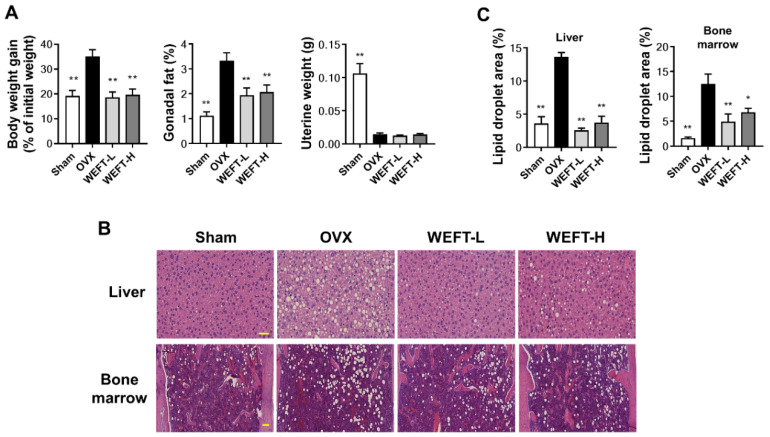
WEFT reduces OVX-induced fat accumulation in live and bone marrow. (**A**) Body weight gain, gonadal fat weight, and uterine weight in each group were measured after 6 weeks of sample administration (WEFT-L, 100 mg/kg/day; WEFT-H, 300 mg/kg/day) following sham or OVX. (**B**) Hematoxylin and eosin staining images of liver or bone marrow tissues (scale bar, 100 µm). (**C**) Histological images of each tissue were randomly selected and analyzed for the quantification of lipid droplets. * *p* < 0.05, ** *p* < 0.01 versus OVX group.

In addition to bone loss, OVX induced an increase in body weight gain, gonadal fat weight (Figure 5A), and fat deposits in the bone marrow and liver (Figure 5B,C) compared to sham-operated mice (*p* < 0.01). This is consistent with a previous study showing that estrogen deficiency deregulates the balance of energy metabolism and increases adipose tissue deposition in bone marrow [29,30]. It has also been suggested that fat accumulation in bone marrow due to the preference of mesenchymal stromal cells to adipocytes over osteoblasts increases fracture risk and decreases fracture repair [31,32]. We found that WEFT significantly ameliorated OVX-induced weight gain and fat accumulation without changes in uterine weight. These results suggest that WEFT might be a useful candidate for the prevention of osteoporosis and fat accumulation in postmenopausal women without hormonal side effects. However, the molecular mechanisms by which WEFT suppresses fat accumulation in tissues should be investigated in further studies.

### 2.4. Phytochemical Profiles of WEFT

**Figure 6:**
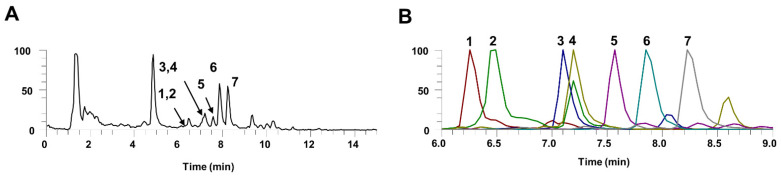
UHPLC-MS/MS analysis of WEFT. (**A**) Base peak chromatogram of WEFT with positive and negative ionization modes on UHPLC-MS/MS. (**B**) Extracted ion chromatogram of identified phytochemicals eluted at 6.0–9.0 min. 1, peimisine glucoside; 2, yibeissine; 3. peiminoside; 4, sipeimine glucoside; 5, peimisine; 6, peimine; 7, peiminine.

High-performance liquid chromatography-tandem mass spectrometry (HPLC-MS/MS) is one of the most accurate and critical analyses for the characterization of active constituents in herb material by providing extensive chemical profiling prior to its isolation and purification [33]. To understand the molecular basis for the bone protective effects of WEFT, we characterized the constituents of WEFT using an ultrahigh-performance liquid chromatography-tandem mass spectrometry (UHPLC-MS/MS) analysis. Alkaloids are the fundamental components of *F. thunbergii* bulbus [12,34]. Seven alkaloids (peimisine glucoside, yibeissine, peiminoside, sipeimine glucoside, peimisine, peimine, and peiminine) were identified in WEFT (Figure 6 and Table 1) by using the retention time and mass spectral data of reference standards and previous studies [35,36]. These alkaloids are characterized by a cevane skeleton, which is composed of an isosteroid structure with a quinolizidine system. It has also been suggested that peiminine shares the same pharmacophore as peimine [37]. Peiminine has been shown to inhibit LPS-induced NF-κB activation and interleukin-1β-induced MAPK activation [38,39]. The activation of NF-κB and MAPK pathways plays key roles in osteoclastogenesis and bone resorption in inflammatory bone loss [40,41]. Recently, it was reported that peiminine suppresses OVX-induced bone loss by inhibiting osteoclastogenesis through the suppression of RANKL-induced NFATc1, ERK, and NF-κB signaling pathways [42]. In this study, we found that WEFT suppressed osteoclastogenesis by inhibiting NFATc1, MAPK (ERK and JNK), and NF-κB signaling pathways. Therefore, the phytochemical constituents of WEFT, including peiminine, may account for the pharmacological activity of WEFT against osteoclastogenesis or OVX-induced osteoporosis, although further studies are needed to elucidate the key active components in WEFT in the near future.

## 3. Materials and Methods

### 3.1. Materials

Cell culture medium, fetal bovine serum (FBS), Enzyme Free Cell Dissociation Solution, and VitD3 were obtained from Thermo Fisher Scientific (Waltham, MA, USA). All antibodies were purchased from Cell Signaling Technology (Danvers, MA, USA), except for β-actin, c-Fos, and NFATc1 from Santa Cruz Biotechnology (Santa Cruz, CA, USA). Recombinant human M-CSF and recombinant GST-tagged human RANKL were obtained as previously described [43]. Reference standards (yibeissine, sipeimine glucoside, peimisine, and peiminine) for UHPLC-MS/MS analysis were obtained from Targetmol (Wellesley Hills, MA, USA). WEFT was purchased from the National Development Institute of Korean Medicine (Gyeongsan, Korea). In brief, WEFT was prepared from *F. thunbergii* bulbus by reflux extraction for 3 h. The extract was filtered and dried in a vacuum freeze dryer.

### 3.2. BMM Preparation

Bone marrow cells were obtained from the bone marrow of the femur and tibia of 7-week-old male C57BL/6J mice. After the ends of both bones were cut using surgical scissors, the bone marrow was removed by PBS flushing using a 1-gauge needle syringe. Red blood cells in the emitted PBS were lysed using RBC lysis buffer for 3 min and then centrifuged for 5 min at 2000 rpm. The collected cells were suspended and filtered using a cell strainer (70 μm), and then the cells were incubated in a cell culture plate with M-CSF (20 ng/mL) for 24 h. Suspended cells were collected and cultured in non-coated cell culture plates for five consecutive days with M-CSF (60 ng/mL). After reaching 90% confluency, BMMs that remained adhered to the plate were dissociated using Enzyme Free Cell Dissociation Solution and then collected using a cell scraper.

### 3.3. Cell Culture

MLO-Y4 cells, a murine osteocyte-like cell line, were cultured in α-MEM supplemented with 2.5% FBS, 2.5% calf serum, and 1% penicillin/streptomycin on type I collagen-coated plates. The passage number of MLO-Y4 cells was maintained at 40 passages (Kerafast, Boston, MA, USA). Phenotypic criteria, including characteristics such as star-shaped with dendritic extensions and low alkaline phosphatase expression for osteocytes, were used to maintain the cell line. BMMs were cultured in α-MEM containing 10% FBS, 1% penicillin/streptomycin, and M-CSF (60 ng/mL).

### 3.4. Cell Viability

BMMs were seeded in 96-well microplates at a density of 2 × 10^4^ cells/well. Twelve hours later, the cells were treated with vehicle or different concentrations of WEFT (11.1, 33.3, 100, and 200 μg/mL) for 1 d. Cell viability was determined by using a CellTiter-Glo luminescent cell viability assay according to the manufacturer’s instructions (Promega, Madison, WI, USA)

### 3.5. Osteoclast Differentiation

For the co-culture differentiation of osteoclasts, MLO-Y4 cells were cultured in a 96-well plate at a density of 1 × 10^3^ cells per well in α-MEM medium containing 10% FBS for 1 day. BMMs (4 × 10^4^ cells/well) were combined with the culture of MLO-Y4, and VitD3 (10 nM) was added as a differentiation inducer in the co-culture plate for 5 days. For the single culture for osteoclast differentiation, BMMs were seeded in a 96-well plate at a density of 1 × 10^4^ cells/well and cultured for four consecutive days in the presence of M-CSF (60 ng/mL) and RANKL (50 ng/mL). Vehicle or different concentrations of WEFT (11.1, 33.3, 100, and 200 μg/mL) were added to each plate on day 0.

### 3.6. TRAP Activity and Staining

TRAP activity was examined by a phosphatase reaction with p-nitrophenyl phosphate as a substrate in a sodium acetate buffer. After formalin fixation and permeabilization, the cells were incubated in a TRAP assay solution (0.05 M sodium tartrate, 120 mM sodium acetate, and p-nitrophenyl phosphate, pH 5.2) for 15 min at 25 °C. When dephosphorylated yellow products were generated by phosphatase, the reaction solution was transferred to a new plate and terminated by adding 0.1N NaOH. The visible color of the reaction product was measured at a wavelength of 405 nm (BD Versamax). After the TRAP activity assay, the remaining cells were stained using naphthol AS-MX phosphate and Fast Red Violet LB salt as a substrate in sodium tartrate buffer (50 mM, pH 5.2). TRAP-positive stained cells harboring more than three nuclei were counted as osteoclasts.

### 3.7. Western Blot Analysis

Total protein lysates were obtained from BMMs using RIPA lysis buffer by repeated vortexing and low temperature-incubation (iNtRON Biotechnology, Sungnam, Korea). Protein concentrations were determined from the absorbance of standard BSA reacted with bicinchoninic acid (BCA) reagent (Thermo Fisher Scientific, Waltham, MA, USA). The total proteins were separated on a 10% SDS-PAGE gel, which was semi-electronically transferred to a polyvinylidene fluoride membrane. To reduce the non-specific binding of antibodies, the membranes were blocked with 5% skim milk for 2 h. Specific primary antibodies (1:1000 dilution) against each target protein and horseradish peroxidase-conjugated secondary antibodies (1:5000 dilution) were incubated with the membranes for at least 1 h. Tris-buffered saline with 0.1% Tween 20 (TBST) washing in-between antibody reactions was performed three times. Chemiluminescent bands at the expected molecular weight were detected using SuperSignal^®^ West Pico Chemiluminescent Substrate under the ChemiDoc Imaging System (Bio-Rad, Hercules, CA, USA). All Western blots were performed in three independent experiments. The protein bands were quantified by densitometry using Image Lab software version 5.2.1 (Bio-Rad, Hercules, CA, USA), and the levels of MAPK (ERK, JNK, and p38) phosphorylation and IκBα degradation were normalized to total each MAPK and β-actin, respectively.

### 3.8. qPCR Analysis

Total RNA was extracted using a spin-column-based nucleotide isolation protocol (iNtRON Biotechnology, Sungnam, Korea) according to the manufacturer’s instructions. RNA purity and concentration were measured using a NanoDrop spectrophotometer (Thermo Fisher Scientific, Waltham, MA, USA). For cDNA synthesis, total RNA (1 μg) was reacted with cDNA reverse transcriptase using a High-Capacity cDNA Reverse Transcription Kit (Thermo Fisher Scientific, Waltham, MA, USA). The generated cDNA was amplified using TaqMan Universal Master Mix II (Applied Biosystems, Foster City, CA, USA) and TaqMan probes for target genes in an ABI 7500 Real-Time PCR Instrument (Applied Biosystems). The TaqMan probes for NFATc1 (Mm00479445_m1), c-Fos (Mm00487425_m1), Tnfsf11 (Mm00441908_m1), Tnfrsf11b (Mm0043542_m1), Csf1 (Mm00432686_m1), MafB, (Mm00627481_s1), Irf8 (Mm00492567_m1), and 18S (Mm99999915_g1) were applied for qPCR reactions in this study. The relative mRNA expression of target genes was calculated by the ΔΔCt method, which is represented as fold change.

### 3.9. In Vivo Study

The Institutional Animal Care and Use Committee (IACUC) at Knotus (Guri, Korea) reviewed the ethnic and scientific judgment of animal experiments and approved the study (approval number: 19-KE-216). Female C57BL/6J mice (6 weeks old) were housed in a temperature-controlled room (22–24 °C, 55% humidity) with an illumination cycle (12 h light/dark cycle) under specific pathogen-free conditions. For OVX operation, animals were anesthetized with a Zoletil/Rumpun mixture, and then the bilateral ovaries were removed through a < 1 cm dorsal incision. A week after OVX or sham operation, healthy mice that recovered from the surgery were selected and randomly assigned into four groups (*n* = 6): sham group + distilled water, OVX group + distilled water, OVX + WEFT 100 mg/kg/day treatment group (WEFT-L), and OVX + WEFT 300 mg/kg/day treatment group (WEFT-H). A commercial normal-fat diet (Research Diet, New Brunswick, NJ, USA) and water were provided ad libitum. For pharmacological studies, distilled water or WEFT was administered once daily by oral gavage for 6 weeks. The body weight of the mice was measured once a week. Body weight change between the final and initial body weight was expressed as a percent change relative to the initial body weight. At the experimental endpoint, the serum levels of CTX-I and PINP were measured using ELISA kits (Immunodiagnostic Systems Ltd., London, UK).

### 3.10. Trabecular Bone Analysis

The distal femurs of the mice were fixed in 10% neutral buffered formalin for 2 days. A µ-CT imaging system (SkyScan 1276, Bruker, Kontich, Belgium) was used for tissue scanning. The original images of the femur were reconstructed and analyzed using SkyScan NRecon and CTAn software, respectively. The volume of interest started at 80 µm below the lower end of the growth plate and extended 1.2 mm in height proximally. Trabecular morphometric parameters including BMD, BV/TV, Tb.N, Tb.Sp, and Tb.Th were calculated.

### 3.11. Histological Analysis

The tissues were fixed in 10% neutral buffered formalin for 48 h at 25°C. After dehydration using a series of ethanol dilutions (70–100%), the tissues were embedded in paraffin and sectioned into a 5 µm thick block. RDO Gold (RDO, Aurora, IL, USA) was used to decalcify bone tissue for 1 week between fixation and dehydration. Tissue sections were stained with hematoxylin and eosin. Lipid droplet areas in representative images were obtained under a light microscope and analyzed using Image J software (National Institutes of Health, Bethesda, MD, USA).

### 3.12. UHPLC-MS/MS Analysis

A Dionex UltiMate 3000 HPLC system assembled with a Thermo Q-Exactive mass spectrometer was used to analyze the WEFT. An Acquity BEH C18 column (100 × 2.1 mm, 1.7 µm) was used for stationary phase chromatography separation. A gradient mixture of 0.1% formic acid in water (A) and acetonitrile (B) was used for the mobile phase, with a flow rate of 0.25 mL/min. The gradient setting was 3% B for 0–1 min, 3–15% B for 1–2 min, 15–50% B for 2–13 min, 50–100% B for 13–20 min, 100% B for 20–23 min, and 3% B for 23.5–27.5 min. The Q-Exactive mass spectrometer equipped with a heated electrospray ionization source was operated in the positive and negative ionization switching modes, according to a previous study [36]. Data acquisition and analysis were performed using Xcalibur v.3.0 and Tracefinder v.3.2 software (Thermo Fisher Scientific, Waltham, MA, USA). The phytochemicals in WEFT were identified by comparing the retention time and mass spectral pattern of reference standards or according to a previous report [35].

### 3.13. Statistical Analysis

In Vitro data are represented as mean and standard deviation, and in vivo data are presented as the mean and standard error of the mean. Group comparisons were performed using a one-way or two-way analysis of variance (ANOVA). Post-hoc analysis was performed with Dunnett’s or Bonferroni’s post hoc test. Statistical significance was set at *p* < 0.05.

## 4. Conclusions

This study is the first to show the potential pharmacological effects of WEFT on menopausal bone health. WEFT inhibited osteoclast differentiation by suppressing RANK signaling and NFATc1 induction in osteoclast precursor cells. In the OVX mouse model, WEFT prevented estrogen deficiency-induced bone loss and weight gain without promoting uterine hypertrophy. Additionally, we identified seven alkaloids in the phytochemical profiling of WEFT that might contribute to the anti-osteoclastogenic or anti-osteoporotic properties of WEFT. Altogether, these results suggest that WEFT is a promising herbal candidate that can be applied to prevent or treat postmenopausal osteoporosis.

## Figures and Tables

**Table 1:** Phytochemicals identified in WEFT by UHPLC-MS/MS.

No	Rt (Min)	Calculated (*m*/*z*)	Estimated (*m*/*z*)	Adducts	Error (ppm)	Elemental Composition	MS/MS Fragments (*m*/*z*)	Identifications
1	6.25	590.3687	590.3686	[M + H]^+^	−0.259	C_33_H_51_NO_8_	590.3686, 572.3564	Peimisine glucoside [35]
2	6.49	444.3108	444.3105	[M + H]^+^	−0.718	C_27_H_41_NO_4_	444.3105, 426.301, 114.0915	Yibeissine *
3	7.10	594.4000	594.3998	[M + H]^+^	−0.444	C_33_H_55_NO_8_	594.3998, 576.3887	Peiminoside [35]
4	7.19	592.3844	592.3840	[M + H]^+^	−0.609	C_33_H_53_NO_8_	592.384, 574.373	Sipeimine Glucoside *
5	7.57	428.3159	428.3157	[M + H]^+^	−0.504	C_27_H_41_NO_3_	428.3156, 410.3038	Peimisine *
6	7.85	432.3472	432.3469	[M + H]^+^	−0.757	C_27_H_45_NO_3_	432.3469, 414.3364	Peimine [35]
7	8.22	430.3316	430.3314	[M + H]^+^	−0.488	C_27_H_43_NO_3_	430.3311, 412.3201	Peiminine *

* Compared with the retention time (Rt) and mass spectral data of reference standards.

## Data Availability

All data of this study are presented within this study.
